# Impact of Helicopter Emergency Medical Services on endovascular thrombectomy delays, clinical outcomes, and societal costs in large vessel occlusion stroke: a retrospective registry based counterfactual simulation study

**DOI:** 10.1186/s13049-026-01658-6

**Published:** 2026-07-08

**Authors:** Axel Ackermann, Sami Curtze, Paulus Torkki, Jukka Pappinen

**Affiliations:** 1 Department of Public Health, Faculty of Medicine, University of Helsinki, P.O. BOX 00020, Helsingin Yliopisto, Helsinki, 00014 Finland; 2 Department of Neurology, Helsinki University Hospital and University of Helsinki, Helsinki, Finland

**Keywords:** Stroke, Large vessel occlusion, Endovascular thrombectomy, Helicopter Emergency Medical Services, Prehospital transport, Counterfactual simulation, Cost-effectiveness, Ordinal shift analysis

## Abstract

**Background:**

The efficacy of endovascular thrombectomy (EVT) for large vessel occlusion (LVO) stroke is highly time-dependent. This study evaluates the logistical, clinical, and economic utility of physician-staffed Helicopter Emergency Medical Services (HEMS) within a centralized stroke network.

**Methods:**

This retrospective study (2021–2025) of 617 EVT-treated LVO patients used spatial simulations on a 159-patient subset, excluding those with missing data, non-transport delays, and distances ≤ 30 km. Simulation 1 compared 42 realized HEMS missions to simulated ground transports across varying on-scene times (OST). Simulation 2 modeled 117 ground-transported patients (> 30 km) as hypothetical HEMS missions under simultaneous vs. secondary dispatch protocols. Clinical and economic impacts were estimated using ordinal logistic regression, HERMES meta-analysis estimates, and a 2-year societal cost model.

**Results:**

HEMS patients had higher baseline severity (median NIHSS 19 vs. 14, *p* < 0.001). Multivariable regression confirmed EVT delay independently predicted a 90-day mRS shift. In Simulation 1, a “scene-time paradox” was identified: prolonged on-scene stabilization negated the aerial transit advantage, with the Rapid-Extrication Scenario showing a modest 1.7-minute HEMS saving whereas the Matched-OST Scenario isolating pure transit speed yielded a 19.7-minute advantage. In Simulation 2, simultaneous HEMS dispatch saved a median of 4.8 min for patients located > 30 km away, with over 31% saving > 20 min. Conversely, secondary dispatch caused a median time loss of 3.8 min. Implementing an optimized simultaneous dispatch protocol could prevent 0.76 to 1.98 cases of severe disability over the 5-year period, equating to 2-year societal savings of €39,000 to €107,000 (annualized ~€7,800 to €21,400 per operational year).

**Conclusions:**

While HEMS provides a transit advantage for LVO stroke, its utility is heavily influenced by on-scene critical care requirements and dispatch timing. Because secondary dispatch negates aerial speed advantages, simultaneous dispatch is essential for maximizing both clinical and economic benefits.

## Introduction

The management of acute ischemic stroke (AIS) is critically time-dependent. The “Time is Brain” axiom quantifies this urgency: in a typical large vessel occlusion (LVO), approximately 1.9 million neurons are lost per minute of untreated ischemia [1]. While intravenous thrombolysis (IVT) remains the cornerstone of AIS treatment, it achieves limited recanalization in LVOs [2], rendering endovascular thrombectomy (EVT) the standard of care in this population [3]. Current European guidelines recommend EVT within 6 h of symptom onset [4], with extended windows up to 24 h in selected patients [5, 6]. However, because the probability of a disability-free outcome declines continuously with every minute of delay, earlier reperfusion is invariably superior [7]. 

In the Helsinki University Hospital (HUS) region, highly optimized in-hospital workflows have reduced the median door-to-needle time to under 20 minutes [8], leaving the prehospital phase as the primary source of reducible delay [9]. Guidelines traditionally recommend transporting AIS patients to the nearest Primary Stroke Center (PSC) for IVT, followed by secondary transfer to a Comprehensive Stroke Center (CSC) for EVT, if endovascular treatment is required (the “Drip-and-Ship” model). However, for suspected LVOs, the “Mothership” strategy (direct transport to a CSC) is favored if the additional travel time is limited, typically under 15 to 30 min [4]. 

Helicopter Emergency Medical Services (HEMS) offer a potential solution for rapid transit, though the evidence from recent systematic reviews remains inconclusive regarding its efficacy. While one meta-analysis indicates that HEMS is associated with significantly better functional outcomes [10], another review concludes that the overall clinical benefit remains ambiguous [11]. Time savings typically materialize only over longer transport distances, usually exceeding 30 to 50 km. Recent data from Sweden demonstrated that HEMS was associated with a 3.6-fold higher likelihood of achieving EVT within 180 min for patients located > 50 km from a CSC [12], and simulation data from Denmark suggest helicopter bypass could save approximately 76 min in onset-to-groin puncture time compared to ground transport [13]. 

This dynamic is further influenced by the challenge of prehospital LVO identification [14]. While exact emergency dispatch algorithms remain non-public, they incorporate key elements of the Finnish Prehospital Stroke Scale (FPSS), such as unilateral motor deficits and gaze deviation. Although their high specificity (91%–94%) minimizes aviation overtriage, a low sensitivity (54%–66%) limits early recognition [15, 16]. Consequently, many true LVO patients fail to trigger a simultaneous HEMS dispatch. Instead, they require on-scene paramedic evaluation before secondary dispatch is considered, adding operational delays that leave significant clinical and economic benefits unrealized [17, 18]. 

However, the interaction between on-scene time (OST), dispatch strategy, and net clinical benefit remains poorly understood. We hypothesized that dispatch timing is the primary determinant of whether HEMS provides a net benefit.

This study aimed to evaluate the overall impact of HEMS for LVO stroke within the Helsinki Uusimaa region (FinnHEMS 10 operational area) in Finland. By analyzing both realized and simulated HEMS transports, we aimed to assess transit speeds, evaluate clinical outcomes across the modified Rankin Scale (mRS) spectrum, and estimate the corresponding societal economic significance.

## Methods

### Study design and setting

This was a retrospective, registry-based, observational cohort study covering the period from January 1, 2021, to December 31, 2025. The reporting adheres to the Strengthening the Reporting of Observational Studies in Epidemiology (STROBE) guidelines [19]. The study was conducted within the HUS regional area, which serves a population of approximately 1.7 million inhabitants and features a diverse geography, ranging from a dense urban capital area to rural municipalities and an archipelago.

Prehospital care is provided by paramedic-staffed ambulances supported by emergency physicians. A single HEMS unit (FinnHEMS 10) is stationed at Helsinki-Vantaa Airport, operating an Airbus EC135 helicopter. In 2024, the unit responded to 2,285 alarms, of which 17% led to hospital transport [20]. HEMS activation is managed by the national emergency response system using an automated response-time model that concurrently dispatches the fastest available unit for predetermined high-priority, time-critical conditions. Although national standardization efforts exist, specific dispatch guidelines are set regionally by the 21 Wellbeing Services Counties and the HUS region, leading to variations in activation criteria. However, ground ambulance remains the default transport modality. A detailed description of HEMS operational characteristics has been published previously [21]. The regional EMS protocol emphasizes early identification of acute stroke symptoms to trigger a high-priority response. A central component of this protocol is the mandatory advance pre-notification from EMS to the receiving hospital [22]. Within the HUS district, transport is centralized: all stroke code patients requiring in-hospital evaluation and reperfusion therapies are transported directly to HUS Meilahti Hospital, the region’s sole CSC providing 24/7 neurological and EVT services.

A review by the ethical committee was not necessary as registry-based retrospective studies do not require one according to Finnish law (Medical Research Act 488/1999, amendments 295/2004, 794/2010). Approval of the study protocol and access to patient records was granted by the Helsinki University Hospital (HUS/326/2026).

### Participant selection and data sources

The study population was identified by linking data from the Helsinki Stroke Quality Registry (HSQR) with prehospital EMS dispatch database (Merlot Medi) using unique personal identity codes. The study cohort was limited to adults (aged ≥ 18 years) with confirmed AIS secondary to an LVO who received EVT (with or without bridging IVT) within 24 h of symptom onset. Patients receiving only IVT or diagnosed with a non-LVO infarction were excluded. For the geospatial simulation analyses, patients with implausible dispatch coordinates (> 300 km from the CSC) and extreme alarm-to-puncture times (> 360 min) were excluded. These massive delays stemmed from diagnostic challenges (e.g., atypical symptoms or secondary EMS visits) rather than transit speeds, and including them would have falsely exaggerated the simulated time savings. The ordinal logistic regression model was performed on the full EVT cohort, as it uses onset-to-treatment time and does not depend on geospatial data.

### Definitions of time intervals

Prehospital timeline variables were constructed by integrating timestamps from both registries. Regarding the onset-to-treatment time (OTT), a strict hierarchy was applied by prioritizing the exact time of symptom onset, followed by time symptoms were first noticed, and finally time last known well. The endpoint for treatment was defined as the groin puncture time for EVT. The total prehospital time for the simulation models was defined as the sum of specific time intervals. For the counterfactual ground transport model, the total time combined the realized dispatch-to-scene and fixed on-scene durations with the simulated driving time to the hospital. For the counterfactual HEMS transport model, the total time consisted of the fixed activation delay, simulated flight times, fixed OST, and flying time to the hospital.

### Geospatial simulation models

Patient incident coordinates were retrieved from Merlot Medi and spatially jittered by 100 m to ensure privacy. To quantify the time difference between transport modalities, each patient’s realized transport time was compared against a simulated hypothetical alternative.

#### Simulation 1: realized HEMS missions

To quantify the precise time advantage provided by the aviation asset, a counterfactual simulation model was applied to the realized HEMS missions with complete geospatial and outcome data. The objective was to estimate the theoretical transit time had these patients been transported by a standard ground ambulance. Since a ground ambulance was the first unit on scene in all cases, the time from dispatch to scene and the time spent on scene were extracted directly from the realized registry data. Consequently, only the driving time from the scene to the hospital was simulated using the Open Source Routing Machine based on OpenStreetMap data [23], reduced by a validated correction factor of 20% to approximate emergency driving conditions [24]. A standardized door-to-puncture delay of 65 min was applied to the simulated transport times, based on local cohort median. Two operational scenarios were evaluated to account for variations in clinical severity and subsequent OST requirements:

Rapid-Extrication Scenario (Severity-Matched Ground OST): The simulated ground transport utilized an OST of 20.3 min, derived from the actual median OST of a severity-matched sub-cohort of 234 ground ambulance patients with admission NIHSS ≥ 15.

Matched-OST Scenario (Methodological Baseline OST): The simulated ground transport adopted the 38.3-minute median OST recorded by the actual HEMS units. This scenario serves as a sensitivity analysis to isolate pure transit time differences, acknowledging that this extended OST reflects both the clinical need for advanced stabilization and the logistical delay of waiting for a simultaneously or secondarily dispatched helicopter. Together, these scenarios bracket the dual role of OST as both a mediator of the HEMS effect and a marker of clinical severity.

#### Simulation 2: potential HEMS benefit for ground patients (> 30 km)

A second, counterfactual simulation was conducted on all ground-transported EVT patients whose incident location was greater than 30 km from the CSC. Counterfactual flights to a PSC were excluded because the regional EMS protocol mandates centralized transport of all stroke code patients directly to the CSC. To estimate the maximum potential benefit, the model assumed 100% HEMS availability, disregarding limitations caused by weather conditions or simultaneous missions. Both scenarios shared the following fixed parameters: a 6-minute HEMS dispatch delay, a cruise speed of 220 km/h, and a standardized 72-minute in-hospital door-to-puncture delay. The two scenarios differed in the timing of HEMS activation and the resulting OST:

Simultaneous Dispatch Scenario: The dispatch center recognizes the LVO immediately and activates HEMS concurrently with the ground ambulance. HEMS flies directly to the scene, spends a standardized 26-minute OST [25], and transports the patient by air to the CSC.

Secondary Dispatch Scenario: The ground ambulance arrives first and initiates on-scene care. After a 15-minute triage interval, paramedics request HEMS activation. Upon helicopter arrival at the scene, a 10-minute patient handover is performed before air transport to the CSC [25]. 

### Clinical outcome and economic modeling (ordinal shift analysis)

The primary clinical outcome was the shift across the full 90-day mRS distribution (0–6). This approach translates time differences into shifts across the full functional outcome distribution rather than dichotomized endpoints. The clinical impact attributable to the time saved (or lost) by HEMS transport was calculated utilizing two predictive models:

Model 1 (Local Cohort Regression): An ordinal logistic regression model derived from the complete case analysis of the local study cohort, adjusted for age, sex, admission NIHSS, bridging IVT status, and pre-stroke mRS. This model calculated a local common Odds Ratio for every 1 min of time saved from symptom onset to groin puncture.

Model 2 (HERMES Meta-analysis): A supplementary sensitivity analysis utilizing the established effect size (OR 1.0029 per minute saved) from the HERMES individual patient data meta-analysis [3]. 

To estimate the economic impact, an ordinal shift analysis was applied. For each patient, the baseline probability distribution of the 90-day mRS was adjusted using the common Odds Ratio corresponding to their individual simulated time savings or delays. The resulting new mRS probability distribution was then monetized using established 2-year societal stroke costs stratified by mRS category from a comparable Nordic healthcare system [26], adjusted for inflation to 2025 price level (multiplier 1.2231) [27]. This conservative 24-month horizon incorporates direct healthcare costs, social care costs, and indirect costs such as productivity losses per mRS category. The specific 2-year cumulative societal costs applied in the economic model were: €40,220 for mRS 0–2; €108,999 for mRS 3; €126,755 for mRS 4; €225,416 for mRS 5; and €39,142 for mRS 6.

### Statistical analysis

Statistical analyses were performed using R software (version 4.5.0; R Foundation for Statistical Computing) in the secure HUS Acamedic environment. Packages utilized included dplyr, tidyr, lubridate, broom, openxlsx, and MASS. Categorical variables are reported as absolute numbers and percentages, and continuous variables as medians with interquartile ranges (IQR). Between-group differences were analyzed using the Mann-Whitney U test for continuous variables and Fisher’s exact test for categorical variables. Statistical significance was established at *p* < 0.05.

### Missing data handling

For the primary clinical outcome analysis (ordinal logistic regression), a complete case analysis approach was utilized. Patients with missing data in the required covariates (onset-to-treatment time, age, sex, admission NIHSS, pre-stroke mRS, or 90-day mRS) were excluded from the regression model. For spatial simulations, patients lacking exact geospatial dispatch coordinates were excluded from their respective simulation sub-cohorts.

## Results

### Baseline characteristics

The initial linked cohort comprised 617 EVT patients (569 ground, 48 HEMS). For the geospatial simulation analyses, 31 ground-transported patients were excluded: 3 with implausible dispatch coordinates (> 300 km from the CSC) and 28 with alarm-to-groin puncture times exceeding 360 min, as these delays were attributable to diagnostic decision-making rather than transport logistics. This yielded a simulation base cohort of 586 patients (538 ground, 48 HEMS). The ordinal logistic regression model was performed on a cohort of 525 patients, as it does not depend on geospatial data and uses onset-to-treatment time rather than alarm-to-puncture time as the predictor. The initial EVT cohort of 617 patients was reduced to 525 after identifying missing data in 90-day mRS (*n* = 50, 8.1%), pre-stroke mRS (*n* = 48, 7.8%), 24 h NIHSS (*n* = 16, 2.6%), admission NIHSS (*n* = 11, 1.8%), and age (*n* = 1, 0.2%).

Compared to the simulation cohort, the 31 excluded patients were significantly younger (median 64 vs. 72 years, *p* = 0.001) and had expectedly longer alarm-to-puncture times (median 522.9 vs. 119.8 min, *p* < 0.001), but did not differ significantly in sex (41.9% vs. 52.7% male, *p* = 0.271), admission NIHSS (11.5 vs. 15.0, *p* = 0.167), 90-day mRS (2.5 vs. 3.0, *p* = 0.747), 90-day mortality (25.0% vs. 15.5%, *p* = 0.344), or distance from the CSC (14.1 vs. 13.3 km, *p* = 0.400).

**Fig. 1:**
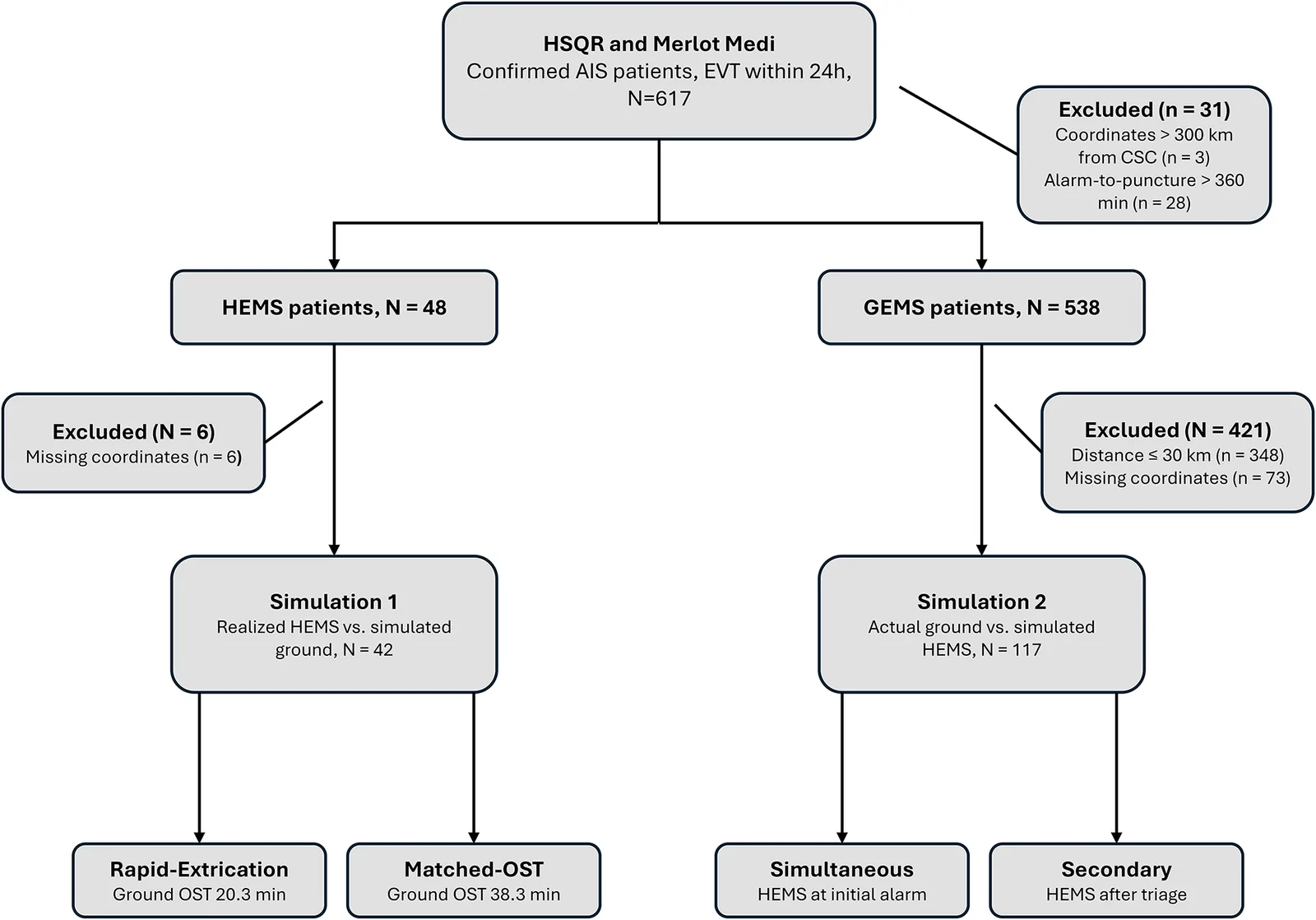
Flowchart of patient selection, exclusion criteria, and simulation cohort formation

For the simulation analyses, 48 HEMS patients entered the Simulation 1 pathway, of whom 6 lacked geospatial dispatch coordinates, yielding a final Simulation 1 cohort of 42. Of the 538 ground-transported patients, 348 were located ≤ 30 km from the CSC and 73 lacked dispatch coordinates, yielding a Simulation 2 cohort of 117 patients located > 30 km from the CSC. Patients excluded due to missing coordinates did not differ significantly from the included cohorts (all *p* > 0.05). The detailed flow of participants is presented in Fig. 1.

Baseline and operational characteristics of the simulation base cohort (*N* = 586) are detailed in Table 1. HEMS patients had significantly higher neurological severity (higher admission NIHSS) and a correspondingly higher 90-day mortality rate. Operationally, HEMS missions involved significantly longer total OST—approximately twice that of ground ambulances—as well as longer transport durations and in-hospital door-to-puncture delays. Despite these severity and logistical differences, the median 90-day mRS did not differ significantly between the groups.

**Table 1:** Baseline characteristics and operational delays of the study population

Characteristic	Ground Ambulance (*N* = 538)	HEMS (*N* = 48)	*P*-value	Missing (*n*)
Male sex, n (%)	297 (55.2%)	29 (60.4%)	0.546	0
Age, years, median [IQR]	72 [61–79]	68.5 [59.8–75]	0.124	1
Pre-stroke mRS, median [IQR]	0 [0–0]	0 [0–0]	0.991	41
90-day mRS, median [IQR]	3 [1–4]	3 [1–6]	0.172	39
Admission NIHSS score, median [IQR]	14 [9–19]	19 [12.8–26.2]	< 0.001	10
24-hour NIHSS score, median [IQR]	7 [2–14]	14 [4–25]	0.001	13
90-day mortality, n (%)	72 (14.3%)	13 (29.5%)	0.015	39
Onset to EMS call, min, median [IQR]	12.3 [5.0–46.8]	7.5 [3.9–18.3]	0.014	0
Total On-scene Time, min, median [IQR]	19.4 [14.9–24.2]	38.3 [21.4–60.4]	< 0.001	84
Total Onset-to-Treatment, min, median [IQR]	142 [113.0–197.5]	177 [133.5–214.0]	0.014	0

Abbreviations: HEMS = Helicopter Emergency Medical Services; EMS = Emergency Medical Services; mRS = Modified Rankin Scale; NIHSS = National Institutes of Health Stroke Scale

Prehospital triage performance and geographic distribution are summarized in Appendix 1.

### Ordinal logistic regression model

Based on the complete case analysis of 525 EVT patients, the multivariable ordinal logistic regression confirmed that every 10-minute delay from symptom onset to EVT was independently associated with lower odds of a favorable functional shift in the 90-day mRS (adjusted OR 0.988; 95% CI, 0.977–0.998; *p* = 0.025). This corresponds to an adjusted common Odds Ratio of 1.0012 for every 1 min of time saved (Model 1). Other significant independent predictors included age, admission NIHSS, pre-stroke mRS, and receipt of bridging IVT (Table 2).

**Table 2:** Ordinal logistic regression model (*N* = 525)

Variable	Unadjusted OR	95% CI	*P* Value	Adjusted OR	95% CI	*P* Value
Age (per 1 year increase)	0.965	0.954–0.977	< 0.001	0.974	0.962–0.986	< 0.001
Female sex	0.861	0.635–1.165	0.332	0.909	0.668–1.237	0.54
Delay to EVT (per 10 min)	0.989	0.979–0.998	0.023	0.988	0.977–0.998	0.025
Admission NIHSS score	0.923	0.903–0.943	< 0.001	0.929	0.908–0.950	< 0.001
Pre-stroke mRS	0.517	0.390–0.678	< 0.001	0.596	0.449–0.788	< 0.001
Bridging IVT administered (Yes)	1.869	1.372–2.551	< 0.001	1.450	1.048–2.008	0.025

Abbreviations: CI, Confidence Interval; EVT, Endovascular Thrombectomy; IVT, Intravenous Thrombolysis; mRS, modified Rankin Scale; NIHSS, National Institutes of Health Stroke Scale; OR, Odds Ratio. The multivariable ordinal logistic regression model assesses predictors for a favorable shift across the 90-day mRS distribution. Complete case analysis was utilized

### Realized benefit of HEMS missions (simulation 1, N = 42)

The counterfactual simulation comparing actual HEMS transports (*N* = 42) against simulated ground scenarios is detailed in Table 3. Under the Rapid-Extrication Scenario (assuming a rapid 20.3-minute OST), HEMS yielded a modest median time saving of 1.7 min, with a slightly negative net economic effect under Model 1 (local cohort) (−€2,225) and a marginal positive estimate under Model 2 (HERMES) (+€1,144). The wide interquartile range (− 57.9 to 19.4 min) reflects the bimodal nature of HEMS missions: patients transported from distant locations with rapid on-scene extrication benefited substantially, whereas short-distance missions involving prolonged physician stabilization resulted in net time losses. When simulated against the Matched-OST Scenario (methodologically matching the 38.3-minute total OST to isolate pure transit speed), HEMS demonstrated a median transit advantage of 19.7 min. In this scenario, half of the simulation sub-cohort saved > 20 min, generating a positive ordinal shift and 2-year societal savings of €1,378 (Model 1, local cohort) to €8,811 (Model 2, HERMES) over the 5-year study period.

**Table 3:** Transport delays, clinical outcomes, and economic impact by simulation and scenario

	Simulation 1: Realized HEMS Missions		Simulation 2: Realized Ground Patients > 30 km from CSC	
	Actual HEMS vs. simulated ground ambulance		Actual ground ambulance vs. simulated HEMS	
**Variable**	**Rapid-Extrication (ground OST 20.3 min)**	**Matched-OST (ground OST 38.3 min)**	**Simultaneous Dispatch (HEMS at initial alarm)**	**Secondary Dispatch (HEMS after paramedic triage)**
	*N* = 42	*N* = 42	*N* = 117	*N* = 117
**1. Transport Times (Alarm to Puncture**,** min)**				
Realized transport time, median [IQR]	162.0 [128.8–186.4]	162.0 [128.8–186.4]	134.6 [116.6–158.6]	134.6 [116.6–158.6]
Simulated alternative, median [IQR]	142.9 [123.9–158.3]	160.9 [141.9–176.3]	127.6 [123.7–131.9]	135.4 [130.7–145.8]
Time saved by HEMS, median [IQR]	1.7 [− 57.9 to 19.4]	19.7 [− 39.9 to 37.4]	4.8 [− 11.1 to 24.8]	−3.8 [− 21.8 to 15.1]
Patients with > 20 min saving, n (%)	10 (23.8%)	21 (50.0%)	37 (31.6%)	23 (19.8%)
**2. Estimated Clinical Impact**				
*Model 1 (Local OR 1.0012/min)*				
Prevented mRS 3–6 cases	−0.21	0.00	0.76	0.36
ARR for mRS 0–2 (%)	−0.5	0.0	0.5	0.2
*Model 2 (HERMES OR 1.0029/min)*				
Prevented mRS 3–6 cases	−0.41	0.07	1.91	0.94
ARR for mRS 0–2 (%)	−1.2	0.0	1.2	0.5
**3. 2-Year Societal Savings (€)**				
Model 1 (Local)	−2,225	1,378	37,795	18,739
Model 2 (HERMES)	1,144	8,811	98,069	51,730

**Simulation 1** compares realized HEMS transports against a counterfactual ground ambulance alternative. The Rapid-Extrication Scenario uses the median OST of severity-matched ground patients (NIHSS ≥ 15), while the Matched-OST Scenario applies the actual HEMS OST to isolate pure transit speed differences. **Simulation 2** models ground-transported patients > 30 km from the CSC as hypothetical HEMS missions. Simultaneous Dispatch assumes HEMS activation at the initial emergency call; Secondary Dispatch models activation after on-scene paramedic assessment. Time saved is calculated as paired individual differences; negative values indicate HEMS was slower. The inflation-adjusted (2025 price level) 2-year societal costs per mRS category were: €40,220 (mRS 0–2), €108,999 (mRS 3), €126,755 (mRS 4), €225,416 (mRS 5), €39,142 (mRS 6), derived from Lekander et al

### Potential benefit for ground-transported patients (simulation 2, > 30 km, *N* = 117)

The hypothetical simulation of 117 ground-transported patients located > 30 km from the CSC is presented in Table 3. The Simultaneous Dispatch Scenario produced a median time saving of 4.8 min, with 31.6% of patients saving > 20 min. Conversely, the Secondary Dispatch Scenario resulted in a median time loss of 3.8 min, indicating the helicopter would have been slower overall. Despite the modest median savings under Simultaneous Dispatch, the substantial time savings in the tail of the distribution shifted the cohort’s overall mRS favorably, leading to potential 2-year societal savings of €37,795 (Model 1, local cohort) to €98,069 (Model 2, HERMES) over the 5-year study period.

### Combined regional potential

Combining the realized benefits of actual HEMS transports (the Matched-OST Scenario) with the theoretical benefits of optimally triaged ground patients (the Simultaneous Dispatch Scenario) illustrates the system’s total potential. A fully optimized regional HEMS approach could prevent 0.76 to 1.98 cases of severe disability over the entire 5-year study period (2021–2025), corresponding to accumulated 2-year societal savings of €39,173 – €106,880 (Model 1 to Model 2).

## Discussion

In this counterfactual simulation, HEMS provided a transit speed advantage for LVO stroke, but the net clinical and economic benefit was heavily dictated by on-scene stabilization requirements and dispatch timing.

A central finding is the “scene-time paradox”—prolonged OST can negate the HEMS speed advantage. Compared to a rapid 20.3-minute ground extrication (the Rapid-Extrication Scenario), actual HEMS missions resulted in only marginal time savings. HEMS patients had higher neurological severity and mortality, and their 38.3-minute median OST reflected both the wait for the helicopter and physician-level stabilization. When the simulation isolated pure transit speed by aligning OST (the Matched-OST Scenario), HEMS yielded a 19.7-minute median time saving. HEMS evaluation must therefore account for this severity confound rather than assuming delayed scene departures are merely inefficiencies.

Dispatch timing critically determines whether HEMS adds value. Simultaneous dispatch demonstrated a positive median time saving, whereas secondary dispatch resulted in an overall median time loss of 3.8 min. Even within the secondary dispatch group, 19.8% of patients saved > 20 min, indicating that for sufficiently remote patients, HEMS provides a net benefit regardless of activation timing. Conversely, realized HEMS missions in our cohort included dispatches within 30 km of the CSC, where archipelago, poor road connectivity, or urban traffic can favor aerial transport despite the short distance. Dispatch decisions should therefore rest on mission-specific predicted time savings rather than rigid distance criteria.

The modest median time saving of 4.8 min under simultaneous dispatch contrasts with the 63-minute savings reported in Swedish rural settings [12] and the 76-minute simulation estimates from Denmark [13], reflecting the shorter transport distances within the HUS region’s single-CSC metropolitan network. However, the median obscures clinically relevant heterogeneity: over 31% of patients saved > 20 min, and this subgroup drove essentially all the observed clinical and economic benefit. HEMS utility in metropolitan regions is therefore not a population-level effect but a targeted intervention for geographically disadvantaged patients.

Currently, the positive predictive value of stroke dispatches is low, and nearly half of HEMS-transported EVT patients were not initially dispatched under a stroke code (Appendix 1), placing them in the slower secondary pathway. This reflects the inherent challenges of remote LVO identification and the known low sensitivity of FPSS-derived criteria [15, 16], underscoring the need for improved dispatcher-level stroke recognition, potentially through AI-assisted dispatch algorithms or telemedicine-guided triage. While identifying every LVO during the initial call remains a theoretical ideal, simultaneous dispatch is already implemented in practice for patients with unambiguous symptoms.

The combined 2-year societal savings for an optimally triaged system were estimated at €39,173 (Model 1) to €106,880 (Model 2) over the 5-year study period. This 2-year horizon captures only a fraction of the true impact: when maintenance-phase costs are projected over the median 7.5-year post-stroke survival, lifetime savings are estimated at €148,000 to €400,000 (annualized €29,000 to €80,000), though this extrapolation is subject to assumptions regarding cost stability and survival. Given that 93% of Finnish HEMS expenses are fixed readiness costs, incorporating LVO patients into the early dispatch framework generates secondary value at negligible marginal cost [28]. The two predictive models used differ markedly in effect size: the local regression (OR 1.0012/min) is substantially more conservative than the HERMES meta-analysis (OR 1.0029/min)^3^, likely because the local cohort includes patients treated over a broader time window (up to 24 h) and adjusts for multiple confounders. The true clinical effect likely falls between these estimates.

### Strengths and limitations

Key strengths of this study include its foundation on a regional cohort of real-world patients with prospectively recorded 90-day mRS outcomes, realized timestamps, and exact geospatial coordinates. The strict inclusion of radiologically confirmed LVO patients undergoing EVT eliminates diagnostic uncertainty. However, this exclusion criterion may simultaneously underestimate the overall benefit of HEMS, as faster aerial transit could have theoretically enabled some excluded patients to arrive within the therapeutic time window for EVT. The bidirectional counterfactual simulation—evaluating both HEMS-to-ground and ground-to-HEMS alternatives within a single cohort—is, to our knowledge, novel. Identifying the “scene-time paradox” as a central determinant of HEMS utility offers a conceptual framework applicable to other systems internationally.

Several limitations must be acknowledged. Residual confounding by indication cannot be fully excluded despite adjustment. The registry data relies on manual documentation, making it susceptible to misclassification of transport modality. The small number of confirmed HEMS missions (*N* = 48) limits statistical power, which is reflected in the wide interquartile ranges observed in the time savings analyses. This heterogeneity arises from the mix of short-distance stabilization and long-distance rapid transport missions within a single cohort; larger, multi-center datasets with more granular timestamps would yield more precise estimates and narrower interquartile ranges. Missing HEMS-specific arrival timestamps prevent separating clinical stabilization from logistical waiting. The exclusion of 31 patients may introduce selection bias, though excluded patients did not differ from the included cohort in functional outcomes or stroke severity. The economic analysis was restricted to a 2-year primary horizon, and the simulations assume idealized HEMS availability. If increased dispatch volumes required expanding fixed operational capacity, these costs would negate the projected societal savings. Finally, the findings are specific to the HUS area physician-staffed HEMS model within a single-CSC network and may not generalize to systems staffed solely by paramedics or those employing a drip-and-ship strategy.

## Conclusions

Physician-staffed HEMS provides a transit speed advantage for LVO patients, but its utility is highly dependent on dispatch timing and on-scene critical care requirements. Secondary dispatch from the scene frequently negates the aerial speed advantage. Optimizing simultaneous dispatch criteria during the initial emergency call offers the greatest opportunity for maximizing the clinical and economic impact of regional HEMS. Future efforts must focus on improving early prehospital LVO detection to ensure the right patients receive rapid aerial transport. 

The positive predictive value of the stroke dispatch code (706) for subsequent EVT was low for both ground (6.2%) and HEMS (16.1%) missions. Prehospital sensitivity for correctly dispatching a stroke code was 74.0% for ground units and 48.9% for HEMS, meaning nearly half of the HEMS-transported EVT patients were initially dispatched under non-stroke codes. Geographically, HEMS dispatches were appropriately concentrated in distant locations, with 94.2% originating beyond 25 km from the CSC, whereas 70% of ground ambulance dispatches occurred within this 25 km zone.

## Appendix 1

**Table:** Prehospital diagnostic performance and geographic distribution

Variable	Ground Ambulance	HEMS
Diagnostic Triage Performance (Stroke Code 706)		
Total 706 missions (n)	6296	155
Sensitivity (%)	74.0	48.9
Positive Predictive Value (%)	63.3	69.7
Ischemic stroke (n)	2623	81
Hemorrhagic stroke (n)	421	19
Stroke mimics (n)	2312	47
Other diagnoses / Unspecified (n)	940	8
Proceeded to EVT (n)	388	25
EVT rate per 706 code (%)	6.2	16.1
Geographic Distribution of Dispatch Incidents		
Distance: 0–25 km, n (%)	4407 (70.0)	9 (5.8)
Distance: 25–50 km, n (%)	770 (12.2)	24 (15.5)
Distance: 50–75 km, n (%)	169 (2.7)	47 (30.3)
Distance: 75–100 km, n (%)	77 (1.2)	35 (22.6)
Distance: 100–125 km, n (%)	15 (0.2)	17 (11.0)
Distance: >125 km, n (%)	35 (0.6)	0 (0.0)

## Data Availability

The datasets used and analysed during the current study are available from the corresponding author on a reasonable request.

## Abbreviations

ARR: Absolute risk reduction
CSC: Comprehensive stroke center
HEMS: Helicopter emergency medical Services
IQR: Interquartile range
mRS: modified Rankin Scale
OR: Odds ratio
OST: On-scene time

## References

[1] Saver JL. Time is brain–quantified. Stroke. 2006;37(1):263–6. 10.1161/01.STR.0000196957.55928.ab. 16339467

[2] Rha JH, Saver JL. The impact of recanalization on ischemic stroke outcome: a meta-analysis. Stroke. 2007;38(3):967–73. 10.1161/01.STR.0000258112.14918.24. 17272772

[3] Goyal M, Menon BK, van Zwam WH, et al. Endovascular thrombectomy after large-vessel ischaemic stroke: a meta-analysis of individual patient data from five randomised trials. Lancet Lond Engl. 2016;387(10029):1723–31. 10.1016/S0140-6736(16)00163-X. 26898852

[4] Turc G, Bhogal P, Fischer U, et al. European Stroke Organisation (ESO) - European Society for Minimally Invasive Neurological Therapy (ESMINT) Guidelines on Mechanical Thrombectomy in Acute Ischemic Stroke. J Neurointerventional Surg. 2023;15(8):e8. 10.1136/neurintsurg-2018-014569. 30808653

[5] Nogueira RG, Jadhav AP, Haussen DC, et al. Thrombectomy 6 to 24 Hours after Stroke with a Mismatch between Deficit and Infarct. N Engl J Med. 2018;378(1):11–21. 10.1056/NEJMoa1706442. 29129157

[6] Albers GW, Marks MP, Kemp S, et al. Thrombectomy for Stroke at 6 to 16 Hours with Selection by Perfusion Imaging. N Engl J Med. 2018;378(8):708–18. 10.1056/NEJMoa1713973. PMC6590673 29364767

[7] Saver JL, Goyal M, van der Lugt A, et al. Time to Treatment With Endovascular Thrombectomy and Outcomes From Ischemic Stroke: A Meta-analysis. JAMA. 2016;316(12):1279–88. 10.1001/jama.2016.13647. 27673305

[8] Meretoja A, Strbian D, Mustanoja S, Tatlisumak T, Lindsberg PJ, Kaste M. Reducing in-hospital delay to 20 minutes in stroke thrombolysis. Neurology. 2012;79(4):306–13. 10.1212/WNL.0b013e31825d6011. 22622858

[9] Puolakka T, Strbian D, Harve H, Kuisma M, Lindsberg PJ. Prehospital Phase of the Stroke Chain of Survival: A Prospective Observational Study. J Am Heart Assoc. 2016;5(5):e002808. 10.1161/JAHA.115.002808. PMC4889170 27139735

[10] Florez-Perdomo WA, Garcia-Ballestas E, Konar SK, et al. Effect of Helicopter Transportation of Acute Ischemic Stroke Patients on Mortality and Functional Outcomes: A Systematic Review and Meta-Analysis. Air Med J. 2022;41(5):476–83. 10.1016/j.amj.2022.07.001. 36153146

[11] Tal S, Mor S. The impact of helicopter emergency medical service on acute ischemic stroke patients: A systematic review. Am J Emerg Med. 2021;42:178–87. 10.1016/j.ajem.2020.02.021. 32089368

[12] Taxbro K, Rashid AS, Skallsjö G, et al. Impact of transport modality on time to endovascular thrombectomy: A population-based registry study in rural Sweden. Scand J Trauma Resusc Emerg Med. 2026;34(1):52. 10.1186/s13049-026-01587-4. PMC12954892 41749283

[13] Behrndtz A, Beare R, Iievlieva S, et al. Can Helicopters Solve the Transport Dilemma for Patients With Symptoms of Large-Vessel Occlusion Stroke in Intermediate Density Areas? A Simulation Model Based on Real Life Data. Front Neurol. 2022;13:861259. 10.3389/fneur.2022.861259. PMC9082641 35547365

[14] Vuorinen P, Kiili J, Alanko E, et al. Cortical symptoms described in emergency calls for patients with suspected large vessel occlusion: a descriptive analysis of 157 emergency calls. BMC Emerg Med. 2022;22(1):146. 10.1186/s12873-022-00706-5. PMC9375237 35962313

[15] Ollikainen JP, Janhunen HV, Tynkkynen JA, et al. The Finnish Prehospital Stroke Scale Detects Thrombectomy and Thrombolysis Candidates-A Propensity Score-Matched Study. J Stroke Cerebrovasc Dis Off J Natl Stroke Assoc. 2018;27(3):771–7. 10.1016/j.jstrokecerebrovasdis.2017.10.015. 29169966

[16] Ollikainen J, Jolma P, Pienimäki JP, et al. Less is more - The Finnish Prehospital Stroke Scale prospective validation. J Stroke Cerebrovasc Dis Off J Natl Stroke Assoc. 2023;32(4):106996. 10.1016/j.jstrokecerebrovasdis.2023.106996. 36801497

[17] Vuorinen P, Setälä P, Hoppu S. Optimizing remote and rural prehospital resources using air transport of thrombectomy candidates. Scand J Trauma Resusc Emerg Med. 2024;32(1):30. 10.1186/s13049-024-01203-3. PMC11022489 38627852

[18] Vaajanen VKE, Vuorinen PET, Setälä PA, Autio R, Hoppu SE. Impact of the first response unit on prehospital on-scene time among paramedic-suspected stroke patients: a retrospective before-after cohort study in Finland. Scand J Trauma Resusc Emerg Med. 2023;31(1):28. 10.1186/s13049-023-01089-7. PMC10265899 37312108

[19] von Elm E, Altman DG, Egger M, et al. The Strengthening the Reporting of Observational Studies in Epidemiology (STROBE) statement: guidelines for reporting observational studies. J Clin Epidemiol. 2008;61(4):344–9. 10.1016/j.jclinepi.2007.11.008. 18313558

[20] Annual report. 2024 [cited 2026 Jan 5]. https://finnhems.fi/finnhems/vuosikertomukset/.

[21] Saviluoto A, Björkman J, Olkinuora A, et al. The first seven years of nationally organized helicopter emergency medical services in Finland - the data from quality registry. Scand J Trauma Resusc Emerg Med. 2020;28(1):46. 10.1186/s13049-020-00739-4. PMC7260827 32471467

[22] Lindsberg PJ. Aivoinfarktin rekanalisaatiohoito - tutkimuksesta kliiniseen käytäntöön. Lääketieteellinen Aikakauskirja Duodecim. 2025;141(21):2095–103.

[23] OpenStreetMap. 2026 [published 2026 Jan 5]. https://www.openstreetmap.org/.

[24] Murray B, Kue R. The Use of Emergency Lights and Sirens by Ambulances and Their Effect on Patient Outcomes and Public Safety: A Comprehensive Review of the Literature. Prehospital Disaster Med. 2017;32(2):209–16. 10.1017/S1049023X16001503. 28134063

[25] Pappinen J, Miettinen T, Laukkanen-Nevala P, et al. The selection of an optimal transportation strategy in urgent stroke missions: a simulation study. Scand J Trauma Resusc Emerg Med. 2020;28(1):48. 10.1186/s13049-020-00747-4. PMC7268427 32487262

[26] Lekander I, Willers C, von Euler M, et al. Relationship between functional disability and costs one and two years post stroke. PLoS ONE. 2017;12(4):e0174861. 10.1371/journal.pone.0174861. PMC5383241 28384164

[27] Statistics Finland [Internet]. [cited 2026 Apr 17]. https://pxdata.stat.fi/PxWeb/pxweb/fi/StatFin/StatFin__khi/statfin_khi_pxt_11xc.px/.

[28] Ackermann A, Pappinen J, Nurmi J, et al. A scenario based approach to optimizing cost-effectiveness of physician-staffed Helicopter Emergency Medical Services compared to ground-based Emergency Medical Services in Finland. Scand J Trauma Resusc Emerg Med. 2024;32(1):60. 10.1186/s13049-024-01231-z. PMC11221128 38956713

